# Systemic Lupus Erythematosus and Deficiencies of Early Components of the Complement Classical Pathway

**DOI:** 10.3389/fimmu.2016.00055

**Published:** 2016-02-24

**Authors:** Ana Catarina Lunz Macedo, Lourdes Isaac

**Affiliations:** ^1^Department of Immunology, Institute of Biomedical Sciences, University of São Paulo, São Paulo, Brazil; ^2^Faculty of Medicine, Children’s Hospital, Clinics Hospital, University of São Paulo, São Paulo, Brazil

**Keywords:** SLE, lupus, complement deficiency, C1q, C2, C4

## Abstract

The complement system plays an important role in the innate and acquired immune response against pathogens. It consists of more than 30 proteins found in soluble form or attached to cell membranes. Most complement proteins circulate in inactive forms and can be sequentially activated by the classical, alternative, or lectin pathways. Biological functions, such as opsonization, removal of apoptotic cells, adjuvant function, activation of B lymphocytes, degranulation of mast cells and basophils, and solubilization and clearance of immune complex and cell lysis, are dependent on complement activation. Although the activation of the complement system is important to avoid infections, it also can contribute to the inflammatory response triggered by immune complex deposition in tissues in autoimmune diseases. Paradoxically, the deficiency of early complement proteins from the classical pathway (CP) is strongly associated with development of systemic lupus erythematous (SLE) – mainly C1q deficiency (93%) and C4 deficiency (75%). The aim of this review is to focus on the deficiencies of early components of the CP (C1q, C1r, C1s, C4, and C2) proteins in SLE patients.

## Systemic Lupus Erythematosus

Systemic lupus erythematous (SLE) is a systemic autoimmune disorder in which multiple autoantibodies against cell nuclear constituents – DNA, histones, and ribonucleoproteins – are produced. The deposition of immune complexes in several organs, mainly kidney, skin, and joints, causes inflammation and tissue damage, producing a broad spectrum of clinical manifestations (1–3) (Table 1) (4). SLE classification criteria was defined by the American College of Rheumatology (ACR) in 1982 (5) and revised in 1997 (6). In 2012, the Systemic Lupus Collaborating Clinics (SLICC) validated the new classification criteria (4). SLE is classified by 4 out of 17 criteria (including at least one clinical and one immunological criterion) or by biopsy-proven lupus nephritis in presence of ANA or anti-dsDNA antibodies (Table 1) (4). Criteria are cumulative and do not need be present concurrently.

**Table 1:** **Clinical and immunological criteria for SLE diagnostic [modified from Ref. (4)]**.

**Clinical criteria**
Acute cutaneous lupus
Chronic cutaneous lupus
Oral ulcers: in the absence of other causes
Non-scarring alopecia in the absence of other causes
Synovitis involving two or more joints, characterized by swelling or effusion OR tenderness in 2 or more joints and 30 min or more of morning stiffness
Serositis in the absence of other causes
Renal: 500 mg of protein/24 h urine or red blood cell casts
Neurologic symptoms in the absence of other known causes such as primary vasculitis, infection, and diabetes mellitus, including toxic-metabolic, uremia, and drugs
Hemolytic anemia
Leukopenia (<4000/mm^3^) or lymphopenia (<1000/mm^3^): in the absence of other known causes
Thrombocytopenia (<100,000/mm^3^) in the absence of other known causes
**Immunological criteria**
Antinuclear antibodies (ANA) titers above laboratory reference range
Anti-dsDNA (double-strand DNA) titers above laboratory reference range. If titer was determined by ELISA consider twice above laboratory reference range
Anti-Sm
Anti-phospholipid antibody: any of the following lupus anticoagulant false-positive rapid plasma reagin (RPR), medium, or high titer of anti-cardiolipin (IgA, IgG, or IgM) or anti-β2 glycoprotein I (IgA, IgG, or IgM) autoantibodies
Low complement: low plasma levels of C3, C4, or CH50
Direct Coombs test positive in the absence of hemolytic anemia

The etiopathogenesis of SLE depends on complement system activation triggered by the presence of immune complexes, leading to inflammation and complement proteins consumption. The acute or continuous low-grade chronic inflammation of lupus often predisposes patients to infections and cardiovascular diseases. Interaction of genetic background with environmental factors makes SLE one of the most complex diseases (7–11). SLE incidence ranges from 1 to 10 per 100,000 person-years and prevalence ranges from 20 to 70 per 100,000. SLE affects both sexes of every age, but predominates in women of child-bearing age with the highest incidence observed in African-Americans. The prevalence for men is approximately 10% of that observed in women (10–12). The disease is extremely rare in children <5 years of age, which demands a more accurate investigation for genetic and immunodeficiency’s causes of SLE. Juvenile systemic lupus erythematous (JSLE – onset before 16 years old) represents 15–20% of all SLE cases and differ in multiple aspects. JSLE is more severe, with higher incidence of nephritis, hematological disorders, photosensitivity, butterfly rash, and mucosal ulceration (13–17).

To study influence of genetic factors, Deapen et al. compared 107 pairs of SLE twins. They found 24% of concordance in 45 pairs of monozygotic while only 6% of concordance in dizygotic twins. This reinforces the importance of genetic predisposition to SLE, though less than first expected, indicating the influence of environmental factors as well (18).

One of the most remarkable genetic associations in SLE is the high frequency of deficiency of the early components of the complement system classical pathway (CP), mainly C1q (90–93%), C1r/C1s (50–57%), C4 (75%), and C2 (10%) (19–22).

## Complement System Activation

The complement system participates in innate and acquired immunity. The proper activation of this system against microorganisms becomes clearly evident in complement immunodeficient patients, who are highly susceptible to recurrent infections.

The complement system is formed by more than 30 proteins present in a soluble form or on the surface of cell membranes. Some proteins act in sequential activation by different pathways: alternative pathway (AP), lectin pathway (LP), and CP. All pathways can lead to activation of a common terminal pathway, generating a membrane attack complex (MAC). This activation is tightly regulated to avoid excessive consumption of complement and tissue injury. Complement proteins may also act as cell surface receptors mediating important interactions with immune cells.

The AP is initiated by hydrolysis of an intramolecular thioester bond located in the C3 alpha chain in the native molecule. Once this bond is broken, the native structural conformation of C3 is modified generating (C3H_2_O), which now exhibits previously hidden binding sites for Factor B leading to the formation of the C3H_2_OB complex. Factor D cleaves Factor B, releasing the fragment Ba generating C3H_2_OBb. This complex has a catalytic site that cleaves C3 into C3a and C3b. C3b has a structural conformation that resembles C3H_2_O and therefore also binds to Factor B (23–27). C3bB is cleaved by Factor D, generating C3bBb (the second C3-convertase) and releasing Ba in the fluid phase. Properdin binds to C3b in this complex and stabilizes C3-convertases of the AP, increasing their half-lives (26, 28–32). Properdin can also bind directly to the surface of certain pathogens (33) and initiates a new platform for deposition of C3-convertase (34–36).

The LP is initiated once lectins, such as mannose-binding lectin (MBL) or ficolins (ficolin-1, ficolin-2, and ficolin-3), bind to microorganisms carbohydrates. MBL-associated serine proteases (MASP 1–3) (37–39) are complexed to MBL or ficolins and they cleave C4 and C2 (40, 41), generating the complex C4b2a (C3-convertase).

The CP is activated mainly by the presence of immune complexes formed by IgM or IgG. However, other proteins, such as C1q, pentraxins, and C-reactive protein, can bind directly to pathogens or other particles and directly activate the CP even in the absence of specific antibodies. Once the immune complex is formed, C1q binds to the Fc portion of the immunoglobulin and immediately activates C1r, which activates C1s. Activated C1s cleaves C4 into C4a and C4b and later C2 already complexed to C4 (C4bC2) generating the C3-convertase (C4b2a), which cleaves C3 into fragments C3a and C3b (42, 43).

All pathways converge to the activation of C3 protein, which can be cleaved by all C3-convertases. C3b fragments can bind to several acceptor molecules including C3-convertases (C3bBbC3b*_*n*_* or C4b2aC3b*_*n*_*) generating C5-convertases, which cleave C5 into C5a and C5b. The next component, C6, binds to C5b, generating C5b6, which now promotes the incorporation of C7 to form C5b67. This complex binds firmly to the cell membrane and later C8 binds to C7 generating C5b678. This complex has lytic activity, which is incremented when several C9 molecules bind to form the MAC – a transmembrane pore (42, 44–46).

## Functions of the Complement System

The activation of the complement system generates active proteins and fragments that play important roles for the innate and acquired immune response (47–49): (a) *production of opsonins*: fragments C3b, iC3b, and C3d bind covalently to acceptor surfaces and molecules and may act as a bridge between pathogens or host cells and complement receptors (CR1 and CR3) present on phagocytic cells. This property may facilitate pathogen killing and antigen presentation complexed to MHC products for T lymphocytes; (b) *production of anaphylatoxins, chemotactic factors, and inflammatory modulators*: fragments C3a and C5a released during complement activation can bind to C3aR or C5aR1 and C5aR2 receptors triggering mast cells or basophil degranulation (50, 51), leading to production and release of important inflammatory mediators. C3a and C5a recruit leukocytes to the inflammatory area. Besides its proinflammatory effects, C3a could act too as inflammatory modulator on neutrophils (52); (c) *increase antibody production*: antigen-bound C3d interacts with CR2 and BCR on B lymphocytes that are activated, proliferates, and produces higher levels of specific antibodies (53). These coated antigens also bind to follicular dendritic cells (DCs), that trap them into germinal centers of lymphoid organs, and display them to B cells to selection of high-affinity B cells (54); (d) *clearance of immune complexes*: fragments C4b and C3b covalently bind to immune complexes. Erythrocyte-CR1 binds to C3b/C4b-coated immune complexes, which are removed from circulation in the spleen and liver; (e) *cell lysis*: the assembly of MAC (C5b6789n) forms a pore in the membrane surface, which leads to osmotic cell lysis; and (f) *removal of apoptotic cells and debris*: C1q (55), C4b, and C3b (56) enhance the ingestion of dead cells by phagocytes (57).

## Pathogeneses of SLE: Inadequate Removal of Debris and Reduction of Self-Tolerance

Apoptosis is a fundamental event for embryogenesis, negative selection of T and B lymphocytes, and maturation of organs. This type of cell death is a coordinated process in which cells undergo cytoskeletal disruption, shrinkage, DNA fragmentation, and plasma membrane blebbing under active control so as not to trigger local inflammation. Production of anti-inflammatory cytokines, such as TGF-beta, after apoptosis is observed (58). Consequently to apoptosis, the cell membrane is modified, and internal phospholipids are exposed on the surface. Opsonization of apoptotic cells is mediated by C1q, C4b, C3b, pentraxins, and collectins. Phagocytic macrophage and DCs remove the opsonized apoptotic debris (58). While apoptosis takes place over a few hours, the removal of apoptotic debris is a very quick process, so as to avoid inflammatory response and activation of T lymphocytes (8). However, apoptotic cells may represent an important source of auto-antigens, breaking self-tolerance and triggering autoimmune diseases.

NETosis is a fast process of cell death in which neutrophils undergoes self-disruption, extruding fibrillary networks composed of DNA, citrullinated histones, and antimicrobial granule peptides [neutrophil extracellular traps (NETs)] that entrap and may kill bacteria, virus, fungi, and protozoan pathogens. In pathological conditions, NETs are associated with tissue damage and autoimmune diseases (7, 59, 60). In lupus, apoptosis and NETosis are deregulated and overactive, providing an overload of self-antigens that under normal circumstances would not be available to be targeted by the immune system. In SLE patients, the clearance of apoptotic bodies is diminished, as is the capability to degrade NETs, related to the reduced DNase I activity (61). The increased load of nuclear auto-antigens could amplify the autoimmune response in SLE patients. Histone post-translational modifications in incompletely cleared apoptotic cells or NETs may generate neoantigens and danger signals with an increased antigenic and immunogenic potential (7, 59, 60).

Systemic lupus erythematous patients commonly present skin photosensitivity. Keratinocytes upon sun exposure undergo apoptosis and expel subcellular blebs, which trigger the production of specific autoantibodies against DNA and nucleosomes. SLE patients also present impairment in the clearance of apototic debris by phagocytic cells (58).

C1q and other proteins, such as immunoglobulins, MBL, serum amyloid P, and C-reactive protein, can influence the adequate removal of apoptotic material, which may be dysfunctional in some SLE patients due to gene polymorphism (7, 59, 60). Macrophages of SLE patients may have defective uptake of apoptotic material (62). The expression of FcγRII and FcγRIII receptors on the monocyte plasma membrane is diminished in monocytes from SLE patients, which may contribute to the impaired clearance of apoptotic debris (63). Complement receptor 1 (CR1 and CD35) present in erythrocytes and other leukocytes is important for the clearance of circulating immune complexes. In SLE patients, the expression of CR1 on erythrocytes is also lower when compared to blood cells from normal individuals. This could be a factor that contributes to a defective clearance of C3b- or C4b-coated immune complexes from the circulation (64).

C1q plays an important role since it binds to apoptotic debris and accelerates the removal of auto-antigens from the immune system (55). Moreover, C1q-deficient and C4-deficient mice present impaired uptake of apoptotic bodies by elicited peritoneal macrophages when compared to wild type control (56). In addition, monocytes not only secrete C1q, C1r, and C1s, as it has at its membrane a functional anchored C1q with globular heads outward that could assemble C1r/C1s, serving to capture apoptotic cells, immune complexes, and pathogen- and danger-associated molecular patterns (PAMPs and DAMPs). Monocytes can also recognize these patterns by cC1qR (receptor for collagen domain of C1q) and gC1qR (receptor for globular domain of C1q) triggering different intracellular signaling pathways (65). Membrane C1q and C1qR on monocytes have been suggested to function as sensing molecules, switching them to DCs or macrophages, depending on the stimuli (66).

Dendritic cells have an important role in preventing autoimmune reactions. The tolerance induction is not fully understood but depends on DC phenotype, type and dose of antigens, cytokine microenvironment, and local synthesis of C1q and its receptors (66, 67). C1q could provide active protection by regulating autoreactive immune cells. Experiments *in vitro* have shown that C1q inhibits T cell proliferation through gC1q/C1q interactions (66, 68, 69).

## Immunodeficiencies of Complement and SLE

According to the European Society for Immunodeficiencies (ESID) registry, deficiencies of complement proteins were responsible for 4.9% (946 out of 19,355) of all primary immunodeficiencies (PID) between 2004 and 2014 (http://esid.org/Working-Parties/Registry/ESID-Database-Statistics). Turley et al. (70), studying 77 complement-deficient patients from the ESID registry in 18 European cities observed that 43% presented defects in the CP, 31% in the AP, and 26% presented defects in terminal complement components. C2 deficiency was the most common of the observed deficiencies (29% of the total). In this series, 37% of patients with defects in the CP have SLE-like disease. These immunodeficiencies were implicated in higher susceptibility to infections: mainly pneumococcal in patients with the CP defects and meningococcal disease in patients with terminal component defects (70).

Since complement-activation products lead to an accentuated inflammatory response in SLE, the disruption of complement activity associated with pathological changes in autoimmune diseases is considered a paradox. Deficiency of early complement components frequently leads to the development of autoimmunity or autoimmune-like manifestations (93% of individuals with C1q deficiency, 60–66% of individuals with C1s–C1r deficiency, 75% of individuals with C4 deficiency, and 10% of individuals with C2 deficiency) (71). Inflammatory and autoimmune diseases were not usually seen in patients deficient in proteins from the terminal pathway. On the on hand, late complement factor deficiencies are preferentially linked to infections and not to autoimmunity (72, 73). On the other hand, homozygous complement deficiency occurs in approximately 1% of SLE patients (74), while 8% of Brazilian and 20% of Indian JSLE subgroup patients have deficiencies of early complement components (75, 76).

## C1q, C1r, or C1s Deficiencies

C1q can recognize a broad range of ligands, from PAMPs to DAMPs, and have been explored as a possible major bridge between innate and acquired immunity. Patients with C1 deficiencies usually present SLE at an early age, in similar female:male proportions, with severe symptoms and prominent cutaneous manifestations (77). Defects in C1 complex proteins are related to point mutations, gene polymorphism, and partial gene deletion (19, 78, 79). Combined deficiencies of C1s/C1r are commonly inherited together. More than 50% of these patients develop SLE. Sixty-seven cases of complete C1q deficiency have been reported (80). More than 90% of patients with homozygous deficiency of C1q are reported to have SLE or lupus-like syndrome. Rash (95%), glomerulonephritis (42%), and alterations in the central nervous system are observed in 18% of C1q-deficient patients. High titers of autoantibodies are observed in more than 70% of these patients (19–21, 78). Successful cure of C1q deficiency was reported with hematopoietic stem cells transplantation in three patients, but one died in consequence of graft-versus-host disease and multi-organ failure (81–83).

## C1q Autoantibodies and SLE

C1q autoantibodies are present in 2–8% of the healthy population, but in SLE, they are present in 30–48% of patients (84). The presence of anti-C1q autoantibodies is accompanied by intense activation of the CP, with very low titers of C1q, C4, and C2 (85). These autoantibodies target a neoepitope of bound C1q that is not expressed in the intact C1 complex (8). This helps to explain the strong association with C1q autoantibodies and nephritis. Their titer correlates to active renal disease with a sensitivity of 44–100% and a specificity of 70–92% (86).

## C2 Deficiency

Homozygous C2 deficiency is more frequent in Western European populations with a prevalence of 1:10,000–20,000, in which the majority (>60%) of these individuals are asymptomatic. Heterozygous C2 deficiency has a frequency of 1–2% in Caucasian populations (19, 87). About 10–30% of homozygous C2-deficient patients develop SLE (19, 88, 89). C2-deficient patients present SLE with a female:male proportion of 7:1. Arthritis, malar rash, discoid rash, and photosensitivity are seen in the majority of C2-deficient patients with SLE (19, 20, 22, 78, 89). The human *C2* gene is located in chromosome 6p21.3. Type I C2 deficiency is caused by a 28-bp deletion in the *C2* gene, which results in the deletion of exon 6 and no translation of the C2 protein. Type II C2 deficiency is usually caused by a point mutation (Ser^189^Phe and Gly^144^Arg), which leads to an impairment in C2 secretion (87, 90), reducing the plasma levels of this protein.

## C4 Deficiency

Complete homozygous deficiency of C4 is rare but is strongly related to SLE. More than 75% of these patients develop this disease. C4 genes are located in chromosome 6p21.3 in the MHC Class III cluster, with two genes encoding C4 protein (C4A and C4B) (91). The gene copy number (CNV) of C4 ranges from 2 to 8. The more common CNV in the healthy population is 2 of each isotype codominantly expressed. SLE is related to reduction of total C4 copy number, but increased number of copies is a protective factor (91, 92). Approximately 50% of SLE-C4-deficient patients develop glomerulonephritis and more than 70% carry ANA and anti-Rho autoantibodies in their serum (19, 20, 78).

The clinical characteristics, prevalence, and presence of autoantibodies in patients with deficiency of early components of the CP are summarized in Table 2 (8, 19–22).

**Table 2:** **Clinical characteristics and prevalence of patients with early complement component deficiencies (8, 19–21)**.

C deficiency	Global prevalence	SLE/SLE-like and complement deficiency	Clinical characteristics	Autoantibody positivity
Clq	Few cases	90–93%	Rash 95%	ANA 75%
			Glomerulonephritis 42%	ENA (Sm, RNP, Ro, and/or La) 70%
			Central nervous system 18%	DNAds antibody 20%
Clr/Cls	Few cases	50–57%	Cutaneous 90%	ANA 75%
			Glomerulonephritis 50%	ENA (Sm, RNP, Ro, and/or La) 70%
C4	Few cases	75%	Glomerulonephritis 50%	ANA 75%
				Anti-Ro 70% (with anti-La negative)
				DNAds antibody 18%
C2	1:10,000–20,000 (homozygous) 1–2% (heterozygous)	10% (homozygous) 2.4–5.8% (heterozygous)	Arthritis 83% Malar rash 92% Discoid rash 67% Photosensitivity 67% Serositis 42%	ANA low titer 25–55% DNAds antibody 33% Anti-Ro 25–50%, 77% (pickering) Anti-histone antibody 20%
Anti-Clq	2–8%	30–60%		

## SLE, Classical Complement Component Deficiencies, and Mortality

Systemic lupus erythematous patients with deficiencies in classical complement components present disease at an early age, with more aggressive symptoms and worse prognosis. Walport et al. (21) reviewed a series of 41 C1q deficiency cases reported in the literature, 38 of which were SLE patients. In these SLE cases, eight individuals younger than 10 years of age died and four of them due of sepsis. Kallel-Sellami et al. (22) reported a 3-year-old girl with SLE and C1q deficiency who died of digestive hemorrhage during treatment of infection. Schaarenburg et al. described 20% fatality in C1q-deficient patients before age of 20 years (93). Jesus et al. (75) studied a total of 72 JLES patients of which six were complement-deficient patients. Three of the six deaths occurred in patients deficient in CP proteins, compared with 1 out of 59 (1.7%) in the group without PID. Two deaths were caused by sepsis, and the third was caused by pneumococcal meningitis. In the same study, disease activity and severity over time, measured by SLICC–ACR damage index, were significantly greater in PID patients (including deficiency of early components of complement and/or immunoglobulins). These descriptions of severe infections in reported cases emphasize the role of the CP in early childhood, when protective antibodies and the anamnestic response have not yet developed. Taken together, these reports reinforce the severity of disease in young SLE patients with ­complement immunodeficiencies of the CP.

## Final Considerations

Systemic lupus erythematous is more severe when associated with deficiencies in components of the CP mainly in young patients. We still do not fully comprehend the real prevalence of complement deficiencies and their association with SLE.

## Author Contributions

All authors listed, have made substantial, direct and intellectual contribution to the work, and approved it for publication.

## Conflict of Interest Statement

The authors declare that the research was conducted in the absence of any commercial or financial relationships that could be construed as a potential conflict of interest.
